# Association of Indiana’s Section 1115 Waiver With Medicaid Enrollment

**DOI:** 10.1001/jamanetworkopen.2019.12922

**Published:** 2019-10-09

**Authors:** Jim P. Stimpson, Yang Wang, Fernando A. Wilson

**Affiliations:** 1 Dornsife School of Public Health, Drexel University, Philadelphia, Pennsylvania; 2 Zilber School of Public Health, University of Wisconsin–Milwaukee, Milwaukee; 3 Matheson Center for Health Care Studies, The University of Utah, Salt Lake City

## Abstract

This cross-sectional study estimates the difference in Medicaid enrollment associated with Indiana’s expansion of Medicaid using a Section 1115 waiver.

## Introduction

The state of Indiana expanded Medicaid February 1, 2015, using a Section 1115 waiver in which the Centers for Medicare & Medicaid Services granted permission to Indiana to charge beneficiaries a monthly premium based on income, charge copayments for nonemergency use of the emergency department, and provide financial incentives for healthy behavior.^1^ We have limited information comparing Medicaid enrollment in states with Section 1115 waivers to states that implemented the full version of Medicaid.^2,3^ The objective of this study was to evaluate how the use of a Section 1115 waiver in Indiana to expand Medicaid was associated with Medicaid enrollment compared with Medicaid expansion states that did not use a waiver.

## Methods

This cross-sectional study included adults aged 19 to 64 years with family incomes at or below 138% of the federal poverty level. States that expanded Medicaid between January 1, 2014, and December 31, 2016, were included in the analysis. States that implemented Medicaid expansion with an approved Section 1115 waiver as of January 2016 (Arkansas, Iowa, Michigan, Montana, and New Hampshire) were excluded from the analysis. We used the 1-year estimates from American Community Survey data provided by the Integrated Public Use Microdata Series.^4^ The date range for the American Community Survey data used was January 1, 2010, to December 31, 2017. State decisions on Medicaid expansion and Section 1115 waivers were linked to Integrated Public Use Microdata Series data using a state identifier code. The final analytic sample size was 1 578 251 respondents. This study was considered non–human subjects research by the Drexel University institutional review board owing to the use of deidentified data and was therefore exempt from requirements for review and participant informed consent. The study followed the Strengthening the Reporting of Observational Studies in Epidemiology (STROBE) reporting guideline.

We used a difference-in-differences linear probability regression to estimate the difference in Medicaid coverage in Indiana associated with the Section 1115 waiver compared with coverage rates if Indiana had expanded Medicaid without the waiver. The state of Indiana was defined as the treatment group. States that expanded Medicaid without a waiver were defined as the control group. Time was defined as either before passage of the Patient Protection and Affordable Care Act (January 1, 2010, to December 31, 2013) or after passage (January 1, 2014, to December 31, 2017). The outcome variable was whether the respondent had Medicaid coverage. Control variables included age, sex, number of children, marital status, poverty status, race/ethnicity, education, employment status, immigration status, and residence in a metropolitan area. All analyses were conducted using Stata/MP statistical software version 15 (StataCorp) and accounted for survey weights and adjusted for state and year fixed effects. Statistical significance was assumed at 2-sided *P* < .05.

## Results

The estimates in the Table indicate that Indiana’s use of a waiver to expand Medicaid was associated with a lower Medicaid coverage rate (−3.5%; 95% CI, −3.501% to −3.499%; *P* < .001) compared with states that expanded Medicaid without a waiver. The parallel trends assumption for the difference-in-differences analysis was met through the regression estimator (*F* = 1.11; *P* = .34).

**Table.** Differences-in-Differences Model for Medicaid Coverage in Indiana Compared With States That Expanded Medicaid Without a Section 1115 Waiver Among 1 578 251 Medicaid-Eligible Adults^a^

Variable^b^	Medicaid Coverage, %
Before Medicaid expansion (2010-2013)	
Control: Medicaid expansion states without a Section 1115 waiver	12.6
Treated: state of Indiana	15.4
Treatment-control difference	2.9^c^
After Medicaid expansion (2014-2017)	
Control: Medicaid expansion states without a Section 1115 waiver	32.9
Treated: state of Indiana	32.3
Treatment-control difference	–0.6^d^
Difference in difference	
After (2014-2017) vs before (2010-2013) difference	–3.5^c^
* R*^2^	14

^a^ Linear probability estimates are based on the sample weights provided by the US Census Bureau. Medicaid eligibility was defined by age 19 to 64 years and income at or below 138% of the federal poverty level. Analyses exclude states that did not expand Medicaid. All years are calendar years (January 1 to December 31). Multivariate adjustment included the following control variables: age, sex, number of children, marital status, poverty status, race/ethnicity, education, employment status, immigration status, and residence in a metropolitan area. Data are from the American Community Survey, 2010 to 2017.

^b^ States that expanded Medicaid between January 1, 2014, and December 31, 2017, without using a Section 1115 waiver were defined as the control group, and the state of Indiana, which expanded Medicaid on February 1, 2015, using a Section 1115 waiver, was defined as the treatment group.

^c^ Statistically significant at *P* < .001.

^d^ Not statistically significant at *P* = .13.

## Discussion

This cross-sectional study found that Indiana’s waiver was associated with negative enrollment gains relative to full Medicaid expansion. Our results should be interpreted within the limitation that our estimates of Medicaid coverage are measured in annual increments that are based on the respondent’s insurance coverage at the time of the survey rather than variation within the year.^5^

Evaluation of Medicaid expansion waivers is complex and requires greater attention as states use these waivers to both expand Medicaid and modify the original intention of the program to increase access to care.^2^ However, it should be emphasized that use of waivers gave states the political leverage to expand Medicaid, which reduced the overall unisurance rate. The potential for a negative impact on Medicaid enrollment relative to full Medicaid expansion should be considered by policy makers, especially in states that have not yet expanded Medicaid or in states that used or are considering using Section 1115 waivers.^3^
